# Binding between Responses is not Modulated by Grouping of Response Effects

**DOI:** 10.5334/joc.233

**Published:** 2022-08-01

**Authors:** Silvia Selimi, Christian Frings, Birte Moeller

**Affiliations:** 1 Cognitive Psychology, University of Trier, Trier, DE

**Keywords:** Action and perception, Event cognition, Action

## Abstract

Several action control theories postulate that individual responses to stimuli are represented by event files that include temporal bindings between stimulus, response, and effect features. Which stimulus features are bound into an event file can be influenced by stimulus grouping. Here, we investigate whether effect grouping moderates response feature binding. For this purpose, we used an adapted response-response binding paradigm introducing a visual effect after each response. These effects could either appear spatially grouped, i.e., close to each other, or non-grouped, thus far from each other. If effect grouping influences response representation, response-response binding effects should be larger for responses producing grouped effects than for responses producing non-grouped effects. In two experiments, we found no indication for a modulation of response-response binding by effect grouping. The role of effect grouping for binding and retrieval processes seems to differ from past evidence regarding stimulus grouping.

## Introduction

Perceptual input is important for action control. Most of the time, we need to perceive objects to interact with them and our interaction in turn results in perceivable effects, be it plucking strings on a guitar that result in different tones, or simply the button presses on your computer keyboard that result in letters appearing on screen. Prinz (e.g., 1992) suggested that perception and action are closely related. The representation of stimulus and response features in partially overlapping neuronal structures allows for interaction of sensory and motor codes without the need of translation from one format to another, an assumption known as the principle of *common coding* (Prinz, 1992, 1997). The common coding principle is a central element in the *theory of event coding* (Hommel et al., 2001; see Shin et al., 2010), which proposes that the representation of a single action integrates codes of response features and perceptual features into one short-term memory trace termed *event file* (Hommel, 2004). Event files have been described as loose networks of binary bindings between individual (stimulus-, response-, and effect-) features of an event (Hommel, 2004), that can, as a central element in the recent *binding and retrieval in action control* framework, account for various classical effects in action control (Frings et al., 2020). While an event file is active, repeating any of the integrated features triggers retrieval of other integrated features, affecting further action. Event files are not limited to one perceptual domain at a time, but were found to include visual, auditory, and tactile information (e.g., Schöpper et al., 2020; Zmigrod et al., 2009). Additionally, these representations are not limited to relevant stimuli, but might also include stimuli that are task irrelevant (Frings et al., 2007), or even the context (Mayr et al., 2018), cognitive control-states (Dignath et al., 2019) and other responses (Moeller & Frings, 2019a, 2019b).

Different factors influence which perceptual information becomes part of an event file and thus is relevant for representation of an action. One factor that was commonly found to influence integration of perceptual information into an event file is grouping. According to the Gestalt principles of grouping, grouped information is perceived as belonging together (Wagemans et al., 2012). Grouping determined whether irrelevant stimuli were integrated into an event-file and thus could retrieve it later on: If an irrelevant stimulus was grouped with a relevant stimulus, the irrelevant stimulus was more likely integrated into the event-file and thus influenced further action (Frings & Moeller, 2012; Frings & Rothermund, 2011; Giesen & Rothermund, 2011; Laub et al., 2018). For example, distractor sounds were only integrated in an event file, if they appeared spatially close to the target stimulus (Moeller et al., 2012).

While these findings indicate an influence of stimulus grouping on event file integration, this was only tested for stimuli present at the time of responding. It is possible that the same also applies to stimuli triggered by responses, i.e. effects. While effects are theorized as part of an event-file, some theories proclaim a special role for them in action representation. For example, the ideomotor principle proposes that actions and effects are so tightly related that the mere anticipation of an effect might suffice to retrieve the action (i.e. the event-file including the motor program) that has been associated with this effect, or, in other words, we represent a response in terms of its perceivable effects (James, 1890; Shin et al., 2010; Stock & Stock, 2004). Here we aimed to analyze whether grouping of effects has a similar impact on integration of information into an event file as has been reported for stimulus grouping. That is, if two effects are grouped, they should be more likely integrated in the same action representation than if they do not appear grouped. In the typical paradigms (e.g., Kunde, 2001; Kunde et al., 2004), different effects are triggered by individual responses. Therefore, we focused on the influence of effect grouping on binding across responses. Through the tight connection between responses and their effects, modulating the relation of two effects might influence how we represent the two actions that triggered these effects. We thus either did or did not group the effects of individual responses and analyzed whether integration of these responses was affected in turn.

To measure binding between individual responses we adapted the response-response (RR-) binding paradigm that was introduced by Moeller and Frings (2019b). In RR-binding, two (or more, Moeller & Frings, 2019a) simple responses are successively planned and executed. Upon execution, these responses are bound to each other in an action representation of higher order, so that a subsequent repetition of one of them retrieves the other and influences execution of this second response. For the present purpose, we introduced a visual effect after each response. These effects could either appear spatially grouped, i.e. close to each other, or non-grouped, thus far from each other.^1^ If effect grouping affects binding and retrieval similar to what is known from stimulus grouping, RR-binding effects should be larger for responses producing grouped effects than for responses producing non-grouped effects.

In two experiments we investigated this question by introducing visual response effects in a grouped vs. non-grouped manipulation. In Experiment 1 effect grouping was varied block-wise while in Experiment 2 effect grouping was varied trial-wise. In an additional control experiment, validating our grouping manipulation (see Appendix A), participants rated to what extent they perceived effects in different spatial positions as being grouped. Anticipating results, we observed standard RR-binding effects but none of the experiments provided evidence for an impact of effect grouping on RR-binding.

## Experiment 1

The aim of Experiment 1 was to investigate whether grouping of effects modulates integration of the corresponding responses. If grouping of effects has an influence on event-file integration, responses that elicit grouped effects should be more likely to be integrated than responses eliciting non-grouped effects. Anticipating that to some degree participants might perceive response effects as artificial, we took measures to increase the perceived relatedness between responses and effects. Previous research found that instructions can influence the way an effect is cognitively represented and can even overrule other influences of response-effect correspondence (Hommel, 1993). We designed the instructions to state that the participants actively make effects light up by giving correct answers. This should prompt the participants to represent the effects in terms of their action goals (making the effect appear; Hommel, 1993). To incentivize participants to attend to the effects, they were also instructed to use them as feedback for whether they answered correctly. To further ensure that responses and effects are perceived as cohesive (Kunde, 2001; Kunde et al., 2004), we presented effects on a horizontal line, similar to the response keys, which are aligned horizontally on the keyboard.

### Method

#### Participants

Thirty students (22 women) from Trier University participated in the experiment. The samples’ mean age was 22 years, with a range from 19 to 35 years. The participants were rewarded with partial course credit. Effect sizes in former studies on RR-binding (computed as t/sqrt(n)) were at least *d* = 0.63 (e.g., Moeller & Frings, 2019b: *d* = 0.63 and *d* = 0.88; Moeller & Frings, 2019c: *d* = 1.07; Moeller & Frings, 2019d: *d* = 0.74 and 1.07). A power-analysis with the program G*Power assuming *α* = .05 and a power of 1–*β* = .85 suggests that at least 25 participants were necessary (Faul et al., 2007).

#### Design

The design comprised three within-subjects factors, namely, effect grouping (grouped vs. non-grouped), response R1 relation (response repetition vs. response change from prime to probe), and response R2 relation (response repetition vs. response change from prime to probe).

#### Materials

The experiment was programmed in PsychoPy3/PsychoJS (2021.1.2; Peirce et al., 2019) and conducted online on Pavlovia (https://pavlovia.org/). For participation, a computer with a physical keyboard was required. Instructions were presented in white [RGB: 255, 255, 255] on a grey background [RGB: 128, 128, 128]. Stimuli were the letters A, B, C, and D and the digits 1, 2, 3, and 4, each with a height of 35 pixels and presented in white. Each display consisted of one letter or digit stimulus presented randomly on one out of 18 positions along an imaginary horizontal line drawn through the center of the screen.

**Figure 1:**
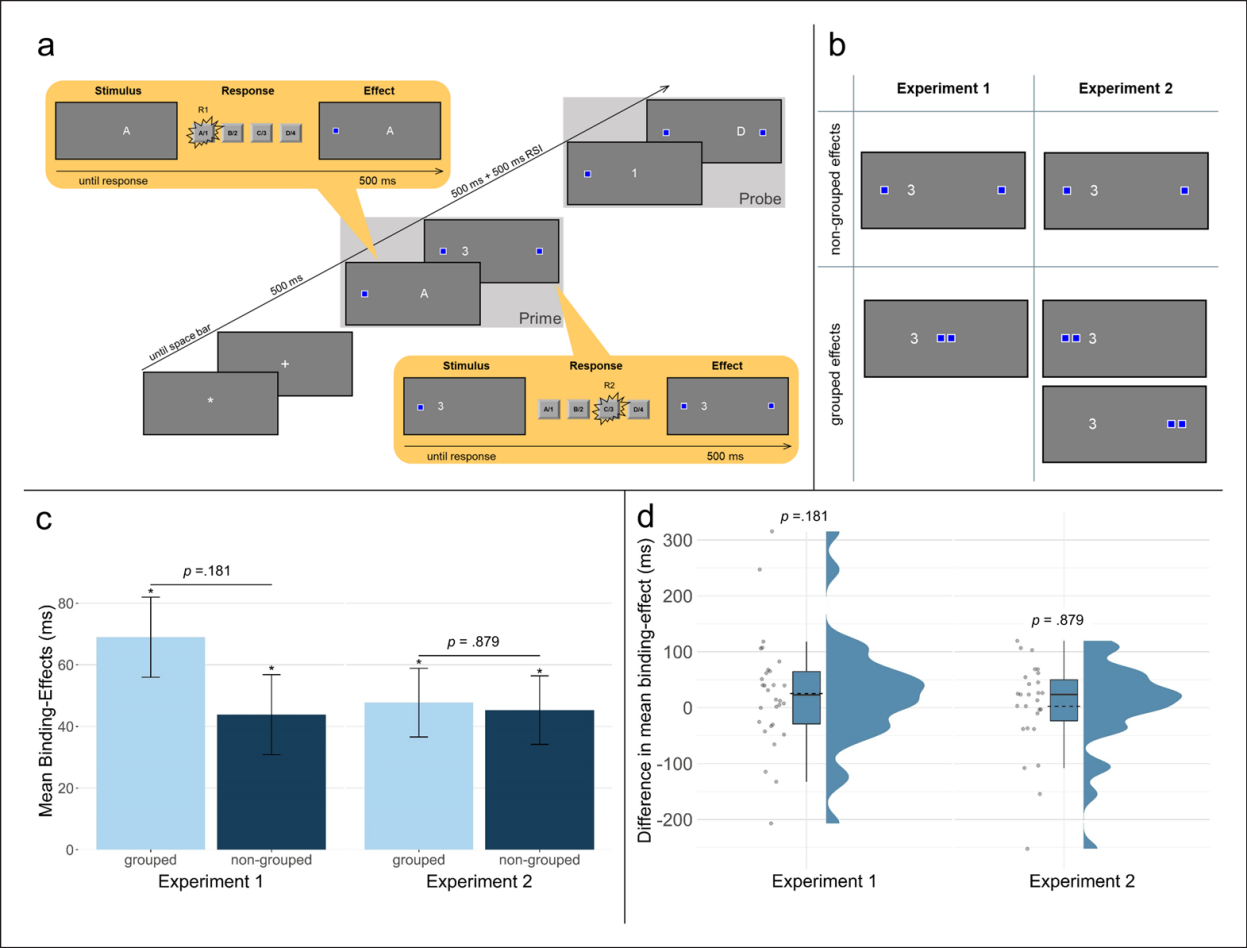
**(a)** Sequence of events in Experiments 1 and 2 in one example trial. Participants gave two successive responses, R1 and R2, both to the prime and to the probe. This is an example of a R1 repetition and R2 change trial in the non-grouped condition. The stimuli and effects are not drawn to scale. **(b)** Effect positions depending on effect grouping condition and Experiment. **(c)** Response-response binding effects in response times across Experiments 1 and 2 as a function of effect grouping (grouped vs. non-grouped). Binding effects were calculated as R1 repetition minus R1 change RTs for R2 change trials, subtracted from R1 repetition minus R1 change RTs for R2 repetition trials [(R1cR2r – R1rR2r) – (R1cR2c – R1rR2c)] **(d)** Distribution of difference in mean response-response binding effects between effect grouping conditions (calculated as [grouped]–[non-grouped] for each participant) for Experiment 1 and 2. Solid lines indicate medians; dashed lines indicate means.

Response effects were signified by blue [RGB: 0, 0, 255] squares with a white border and appeared on one of four positions on the same imaginary center screen line, depending on the condition (coordinates in pixels, center of screen has coordinates [0, 0]: [–530, 0] and [530, 0] for non-grouped condition, and [–30, 0] and [30, 0] for grouped condition). Prime response effects disappeared after the prime, resulting in a maximum of two response effects visible at a time (see Figure 1a & b).

#### Procedure

Before the experiment, participants gave informed consent regarding the recording of personal data and responses during the experiment and indicated their age and gender. Instructions were given on the screen. Participants were instructed to place their middle and index fingers on the keys D, F, J, and K. Each key corresponds to a letter and a digit (A/1, B/2, C/3, and D/4).

Their task was to press the key corresponding to the individually presented letters and digits. Each trial was started by pressing the space bar while an asterisk was presented in the middle of the screen (see Figure 1a). Then a plus sign appeared for 500 ms, followed by the first prime stimulus (letter or digit). Upon correct responses, a first response effect square lit up for 500 ms, upon incorrect responses, the trial continued without a response effect square appearing. Then the second prime stimulus appeared indicating prime response R2. Again, execution of a correct response resulted in the presentation of a second effect square for 500 ms while the response stimulus remained on screen. The position of response effect squares depended on condition (grouped vs. non-grouped, see Figure 1b). Afterwards, a blank screen appeared for 500 ms and was followed by the probe. The procedure in the probe was identical to that in the prime. Every 48 trials participants were allowed to take a short break, after which they resumed the task in their own time.

The relation of R1 between prime and probe (repetition vs. change) was varied orthogonally to the relation of R2 (repetition vs. change). In R1 repetition trials (R1r), the same response was required to the stimulus indicating prime response R1 and the one indicating probe response R1. In R1 change trials (R1c), different responses were required to the stimulus indicating prime response R1 and the one indicating probe response R1. In R2 repetition trials (R2r), the same response was required to the stimulus indicating prime response R2 and the one indicating probe response R2. In R2 change trials (R2c), different responses were required to the stimulus indicating prime response R2 and the one indicating probe response R2. The factor effect grouping was varied block-wise with one block in each of the two conditions. The order of blocks was balanced across participants. Each experimental block included 96 trials, with 24 of each of the four conditions R1rR2r, R1rR2c, R1cR2r, R1cR2c. Stimuli indicating the R1 and R2 responses in prime and probe were selected at random, but in accordance to the requirements of the current condition. There were no stimulus repetitions within a trial. At the beginning of the experiment, participants passed a general practice block introducing both grouping conditions to avoid block order effects (4 trials). Before each experimental block started, they practiced their task for 16 trials (subsample of the experimental trials).

### Results

For the analysis of response times (RTs) we only included trials with correct responses R1 and R2 in both prime and probe. The rate of prime response errors (R1 or R2) was 12.9%. The probe error rates were 6.3% for R1 and 6.6% for R2 (only including trials with correct previous responses). Furthermore, we excluded RTs of more than 1.5 interquartile ranges above the third quartile of the probe R2 RT distribution of the participant (Tukey, 1977) and RTs shorter than 200 ms from the analysis. Due to these constraints, 26.0% of the trials were excluded from the RT analyses. For the mean RTs and error rates, see Table 1.

**Table 1:** Mean response times (in milliseconds) and mean error rates (in percentages) for probe responses R2, as a function of effect grouping, R1 relation and R2 relation between prime and probe.


	GROUPED EFFECTS		NON-GROUPED EFFECTS	
	
	*R2 REPETITION*	*R2 CHANGE*	*R2 REPETITION*	*R2 CHANGE*

*R1 change*	692 (9.1)	628 (2.4)	708 (9.6)	650 (5.4)

*R1 repetition*	668 (7.9)	672 (8.2)	690 (8.2)	676 (5.6)


The dependent variable of interest was performance in probe R2. If prime R1 and R2 are integrated, repeating prime R1 in the probe should trigger retrieval of the second prime response and thus influence performance in probe R2. In a 2 (R1 relation: repetition vs. change) × 2 (R2 relation: repetition vs. change) × 2 (effect grouping: grouped vs. non-grouped) analysis of variance (ANOVA) on probe R2 RTs, the main effect for R2 relation was significant, *F*(1, 29) = 41.35, *p* < .001, *η_p_^2^* = .59, while the main effect for R1 relation was not, *F*(1, 29) = 2.59, *p* = .118, *η_p_^2^* = .08. Additionally, the main effect for effect grouping was significant, *F*(1, 29) = 4.59, *p* = .041, *η_p_^2^* = .14, with longer RTs in the non-grouped than in the grouped condition. More importantly, the two-way interaction of R1 and R2 relation was significant, *F*(1, 29) = 29.01, *p* < .001, *η_p_^2^* = .50, indicating binding between the responses: The repetition of R1 facilitated performance only if R2 was repeated as well, *t*(59) = 4.03, *p* < .001, but impaired performance if R2 changed, *t*(59) = –4.98, *p* < .001. However, this was not further modulated by effect grouping, *F*(1, 29) = 1.88, *p* = .181, *η_p_^2^* = .06, (see Figure 1c, for distributions of participants binding effects differences between grouping conditions, see Figure 1d). Bayes factors provided anecdotal evidence for an absence of the effect grouping modulation, *BF_01_* = 2.21.

In the same analysis on error rates, the main effect of R2, *F*(1, 29) = 15.36, *p* < .001, *η_p_^2^* = .35, was significant, while the main effect of R1, *F*(1, 29) = 2.23, *p* = .146, *η_p_^2^* = .07, was not. However, the interaction of R1 and R2 was significant, *F*(1, 29) = 11.14, *p* = .002, *η_p_^2^* = .28, again indicating binding between the responses: The repetition of R1 did not facilitate performance if R2 was repeated as well, *t*(59) = 1.36, *p* = .18, but impaired performance if R2 changed, *t*(59) = –3.05, *p* = .003. The relation was not further modulated by effect grouping, *F*(1, 29) = 2.82, *p* = .10, *η_p_^2^* = .09, *BF_01_* = 1.47. Taken together, results from both, RT and error rate data, indicate that RR-binding effects are not modulated by grouping of response effects.

### Discussion

Results from Experiment 1 indicate that responses are integrated and thus, they clearly replicate previous findings on RR-binding. However, RR-binding effects were not modulated by effect grouping. Three factors might explain the results: Firstly, it stands to question whether the grouping manipulation itself was actually successful, i.e., whether the participants perceived the two effects as more grouped in the spatially close condition than in the far condition. We used a grouping manipulation for our effects that was similar to the one used before to investigate stimulus grouping on binding and retrieval processes (e.g., Frings & Rothermund, 2011; Moeller et al., 2012). Yet, it is unclear whether such a modulation is perceived the same way when used on response effects. Thus, we conducted a manipulation check experiment (see Appendix A), where participants rated the perceived grouping between response effects. The results were very clear and indicated that participants perceived the effects as significantly more grouped in the spatially close condition than in the far condition. Secondly, one can argue that grouping manipulations might rely on a subjective frame of reference: To perceive two effects as grouped, we need to establish a stable representation of what grouped means in comparison to non-grouped. Since we manipulated grouping block-wise, variance on the factor effect grouping may have lacked to draw sufficient attention to it and to provide a constant comparison of grouped effects for the non-grouped trials and vice versa (for a similar argument regarding the influence of perceptual grouping of stimuli - via figure ground segmentation - on binding, see Frings & Rothermund, 2017). Even though we shortly introduced both conditions at the beginning of the experiment through a general training, this might not have been sufficient to ensure an ongoing representation of the grouped vs. non-grouped manipulation. Thus, introducing a trial-wise manipulation might help establish a proper frame of reference regarding the distance of effects. Thirdly, we cannot be entirely sure that effects were perceived as being related to the responses rather than just being perceived as random. Here a manipulation check would be necessary.

## Experiment 2

In Experiment 2, conditions were varied trial-wise instead of block-wise. To avoid additional complexity of the display with changing effect positions in a trial-wise manipulation, we decided to adjust the effect positions, so that the same four possible positions were used in both conditions. Additionally, we ran a short manipulation check questionnaire at the end of the experiment asking about the participants’ impression on the relatedness between responses and effects.

### Method

#### Participants

Twenty-seven students (20 women) from Trier University participated in the experiment. The samples’ mean age was 23 years, with a range from 19 to 36 years. The participants were rewarded with partial course credit. Three additional participants were excluded due to extremely high error rates (more than 90% of trials had to be excluded).

#### Design

The design comprised three within-subjects factors, namely, effect grouping (grouped vs. non-grouped), response R1 relation (response repetition vs. response change from prime to probe), and response R2 relation (response repetition vs. response change from prime to probe).

#### Materials and procedure

Materials and procedure were identical to those in Experiment 1, except for the following differences. Unlike Experiment 1, the factor effect grouping (grouped vs. non-grouped) was variated trial-wise. Thus, the part of the training introducing the two conditions separately was omitted. Additionally, response effects appeared in one of four possible positions (in pixels, screen center has coordinate [0, 0]: [–540, 0], [–480, 0], [480, 0] and [540, 0], see Figure 1b). For the grouped condition, response effects in each trial appeared in either the two left side or the two right side positions, while response effects in the non-grouped condition always appeared on opposite screen side positions, while maintaining a fixed distance (either [–540, 0] and [480, 0], or [–480, 0] and [540, 0]). At the end of the experiment, participants had to fill out a short questionnaire (six items; see Appendix B) judging whether they perceived their responses and the effects as related (forced choice; 4 items) and rating the strength of that relation (7-point rating scale; 2 items).

### Results

On a questionnaire regarding the perceived relation of responses and effects, the majority (79.8%) of participants reported perceiving the effects as being related to the responses^2^ and rated the strength of this relation with *M* = 5.75 (*SD* = 1.25) on a seven-point scale with 1 being not related and 7 being strongly related. Furthermore, the strength of perceived grouping and the difference in binding effects between both conditions (calculated as [grouped]-[non-grouped] for each participant) did not correlate significantly, *r*(23) = –.07, *p* = .736 (7-point scale), and *r*(23) = –.04, *p* = .842 (forced choice items).

For the analysis of RTs, we considered only trials with correct responses R1 and R2 in both prime and probe. The error rate for prime responses (R1 or R2) was 10.6%. The probe error rates were 5.7% for R1 and 5.1% for R2 (only including trials with correct previous responses). Due to the same constraints as in the previous experiments, 20.0% of the trials were excluded from the RT analyses. For the mean RTs and error rates, see Table 2.

**Table 2:** Mean response times (in milliseconds) and mean error rates (in percentages) for probe responses R2, as a function of effect grouping, as well as R1 relation and R2 relation between prime and probe.


	GROUPED EFFECTS		NON-GROUPED EFFECTS	
	
	*R2 REPETITION*	*R2 CHANGE*	*R2 REPETITION*	*R2 CHANGE*

*R1 change*	677 (7.4)	619 (2.1)	675 (8.5)	622 (3.9)

*R1 repetition*	664 (6.0)	653 (4.5)	654 (6.3)	646 (6.5)


In a 2 (R1 relation: repetition vs. change) × 2 (R2 relation: repetition vs. change) × 2 (effect grouping: grouped vs. non-grouped) ANOVA on probe R2 RTs, the main effect for R2 relation was significant, *F*(1, 26) = 55.85, *p* < .001, *η_p_^2^* = .68, while the main effect for R1 relation was not, *F*(1, 26) = 1.58, *p* = .220, *η_p_^2^* = .06. Additionally, the main effect of effect grouping was not significant, *F*(1, 26) = 1.02, *p* = .321, *η_p_^2^* = .04. More importantly, the two-way interaction of R1 and R2 relation was significant, *F*(1, 26) = 24.72, *p* < .001, *η_p_^2^* = .49, indicating binding between the responses: The repetition of R1 facilitated performance only if R2 was repeated as well, *t*(53) = 3.49, *p* < .001, but impaired performance if R2 changed, *t*(53) = –4.29, *p* < .001. However, this was not further modulated by effect grouping, *F*(1, 26) = 0.02, *p* = .879, *η_p_^2^* < .01, (see Figure 1c, for distributions of participants binding effects differences between grouping conditions, see Figure 1d). This is supported by a Bayes factor of *BF_01_* = 4.86, indicating that the data are more than four times more likely under the null hypothesis that assumes no modulation by effect grouping than under the alternative hypothesis.

In the same analysis on error rates, the main effect of R2, *F*(1, 26) = 9.67, *p* =.004, *η_p_^2^* = .27, was significant, while the main effects of R1, *F*(1, 26) = 0.45, *p* = .507, *η_p_^2^* = .02 and effect grouping, *F*(1, 26) = 3.83, *p* = .061, *η_p_^2^* = .13, were not. However, the interaction of R1 and R2 was significant, *F*(1, 26) = 12.15, *p* = .002, *η_p_^2^* = .32, again indicating binding between the responses: The repetition of R1 facilitated performance if R2 was repeated as well, *t*(53) = 2.00, *p* = .050, but impaired performance if R2 changed: *t*(53) = –3.31, *p* = .002. The relation was not further modulated by effect grouping, *F*(1, 26) = 0.16, *p* = .69, *η_p_^2^* < .01, *BF_01_* = 4.55. In sum, results from RT and error rate data do not indicate modulating effects of response effect grouping on RR-binding effects.

### Discussion

We again replicated binding between responses but found no modulation by grouping of response effects. Introducing a trial-wise instead of a blocked manipulation to establish a frame of reference regarding grouping did neither impact RR-binding nor the grouping manipulation. This was the case, even though results from a manipulation check questionnaire suggest that participants indeed perceived responses and effects as related in the present experiment. This again indicates that grouping of effects has no influence on whether they are integrated in the same action representation.

## General Discussion

In two experiments, we investigated the role of effect grouping on event-file integration. Using an adapted RR-binding task (Moeller & Frings, 2019b), we manipulated whether responses produced grouped vs. non-grouped effects. If grouping influenced whether effects are integrated in the same representation, we expected grouped effects to lead to stronger RR-binding than non-grouped effects. In sum, we could replicate standard RR-binding effects in both experiments. However, these remained unaffected by the effect grouping manipulation. For an overview of binding effects across both experiments, see Figure 1c.

Regarding the impact of grouping on integration of information into event files, time of appearance seems to make a difference: While stimulus grouping seems to affect what is integrated into an event file, effect grouping does at least not affect integration and retrieval of corresponding responses. When considering potential stimuli to interact with, it makes sense to be somewhat selective, as not every stimulus in our environment is relevant for the action we want to conduct. There may also be some stimuli that are irrelevant for the action itself, but are nevertheless integrated into an event file because they are close to relevant stimuli (Frings et al., 2007; Frings & Rothermund, 2011; van Dam & Hommel, 2010) and need to be attended to be avoided, for example, reaching over a line of mugs on a shelf to fetch the bottle behind them (Moeller & Frings, 2014). In contrast, the number of effects that our actions can elicit in ourselves and in the environment is limited and much more relevant for us (to be able to learn and manipulate our environment). Thus, it might not be necessary to be as selective; on the contrary, being selective would here limit our potential to meaningfully interact with our environment. Hence, we do not need to rely on grouping when it comes to effects.

A limitation of our study is that we only manipulated one type of effect. Response effects can be differentiated into body-related effects, e.g. proprioceptive consequences of responses like the sensation of a keypress, and environment-related effects, like stimuli lighting up on screen (Pfister, 2019). These different types of effects co-occur and thus, either of these can be part of an action representation. In our experiments, we only manipulated grouping of environment-related effects. However, body-related effects always remained the same and likely remained grouped, as fingers giving responses were positioned closely together on the keyboard, making the keypress sensations spatially close to each other. While the integration of environment-related effects alone was not influenced by grouping, it remains to be tested whether the same is true for body-related effects. One could even argue that grouping of body-related effects might have overshadowed the grouping manipulation of environment-related effects. In line with this, we find significant RR-binding effects across both conditions in both experiments. Another argument for some kind of overshadowing of environment-related effect grouping by body-related effects comes from comparing the role of effects in action representation in the context of learning. Environment-related effects might only become important for action representation after their relation to responses has been learned, whereas body-related effects are already past early stages of learning (see James, 1890; Pfister, 2019). It remains to be seen whether findings on grouping can be generalized over both, body-related and environment-related effects, or whether these two are affected differently by grouping and how this might be affected by learning.

In this study, we focused on the spatial grouping of response effects, while keeping temporal factors constant. Due to the trial structure one could argue that responses (and effects) might also be temporally grouped: the stimulus indicating the second prime (probe) response followed immediately upon execution of the first prime (probe) response, while there was a 500 ms blank interval after the second prime response before the probe started. There are findings suggesting that the intent to execute two responses as one (temporally grouped) vs. in sequence influences whether they were integrated as one event (Fournier & Gallimore, 2013). Although responses in our paradigm were executed separately, we cannot rule out that temporal grouping of prime and probe responses might have influenced our results. Interestingly, emphasizing the temporal grouping of responses by elongating the time interval between prime and probe does not lead to stronger binding effects (Moeller & Frings, 2021). In the future, it could be interesting to further investigate the temporal relation between responses (and their effects), especially as it has been shown that temporal features like presentation times of stimuli or response-effect time intervals can also be integrated into event files (Bogon et al., 2017; Dignath et al., 2014). Temporal grouping of responses might potentially interact with, or even overshadow, spatial grouping.

Also regarding temporal grouping, a difference from previous studies on stimulus grouping in binding is that in most instances, stimuli shared a common onset (Frings & Rothermund, 2011; Giesen & Rothermund, 2011; Schmalbrock et al., 2022; but see Laub et al., 2018), while in our study, the effect onsets were asynchronous, as they were dependent on response execution. We attempted to alleviate this asynchrony by making the effect of R1 stay on screen during the performance of R2 and the consequent R2 effect presentation. That is, even though the effects differed in their onset time, they were presented together for 500 ms and shared a common offset. Due to these factors, and regarding the additional experiment checking our grouping manipulation (see Appendix A), we are confident that effect stimuli were perceived as grouped at least to some degree. However, we cannot exclude the possibility that the lack of a common effect onset interfered with a grouping perception.

To conclude, grouping of effects does not seem to influence which information is integrated into one action representation. From the results at hand, we can draw two possible conclusions. It might be that the representation of the two responses was altered through their effects but did simply not affect response-response binding. This could be due to overshadowing by temporal grouping factors. Alternatively, it might be that the spatial distance of this kind of response effect did not alter response representation. Here it is possibly important to differentiate between body- and environment-related effects (see Pfister, 2019). For example, a longer learning history might be necessary before the features of a certain effect can affect the representation of the associated response.

## Data Accessibility Statement

Data of this study is available under: https://doi.org/10.23668/psycharchives.5356.

## Additional File

The additional file for this article can be found as follows:

10.5334/joc.233.s1 Appendices. Appendix A and B.

## Footnotes

We used the term ‘grouping’ not as spatial proximity in relation to other elements (see Wagemans et al., 2012), but in a sense that was implemented in studies on binding and retrieval processes in the past (e.g., Frings & Rothermund, 2011; Moeller et al., 2012).

Mean across four forced choice items. Frequency distributions did not differ between items, Χ*^2^*(3) = 3.04, *p* = .385.
