# Supplementary material for: Binding between Responses is not Modulated by Grouping of Response Effects
Source: J Cogn. 2022 Aug 1;5(1):42. doi: 10.5334/joc.233 (PMC9400605; doi:10.5334/joc.233)
Supplement: Appendices. — Appendix A and B. [file joc-5-1-233-s1.pdf]

APPENDIX A: Grouping manipulation-check experiment

To see whether participants would describe close effects in our setup as being more grouped than distant effects, we conducted an additional manipulation-check experiment. There, we combined a shortened version of the response-response binding task (only prime displays were presented) with a subsequent judgment task at the end of each trial.

Methods

Participants. Ten students from Trier University took part in the online experiment. The median age was 22 (range: 19-26) and participants were rewarded with partial course credit.

Design. Participants rated perceived grouping of response effects. The design comprised one within-subjects factor, namely, the distance of response effects (close vs. far).

Materials and procedure. The setup was identical to Experiment 2, except for the following differences: In each trial, only two consecutive responses (R1 and R2) were given instead of four. Again, each response elicited a response effect. The effect positions depended on the distance condition, which was varied trial-wise. Participants completed 4 training trials, followed by 24 experimental trials (12 in each condition). Note that the close condition corresponds to the grouped condition of Experiment 2, while the far condition corresponds to the Experiment 2 non-grouped condition. At the end of each trial, participants performed a rating task judging to what degree they perceived the effects as grouped. Answers were given on a 10-point rating scale with 1 being “not grouped” and 10 being “grouped”. The instructions for the letter/digit identification task and the description of effects remained the same as

in Experiment 2. Regarding the instructions of the rating task, the concept of grouping was introduced with an example that was unrelated to the experimental manipulation, using similarity in arrays of letters. This allowed the participants to set their personal baseline in regards to what they perceived as grouped and what not.

Results and Discussion

Results in the rating-task indicate that the rating of perceived grouping differed significantly between conditions with effects positioned spatially close and spatially distant, $t(9) = 6.11$, $p < .001$, $BF_{10} = 176.24$. Overall, participants reported higher perceived grouping in trials with spatially close effects, ($M = 8.33$, $SD = 1.47$), than with distant effects ($M = 2.92$, $SD = 1.60$, see Figure A1).

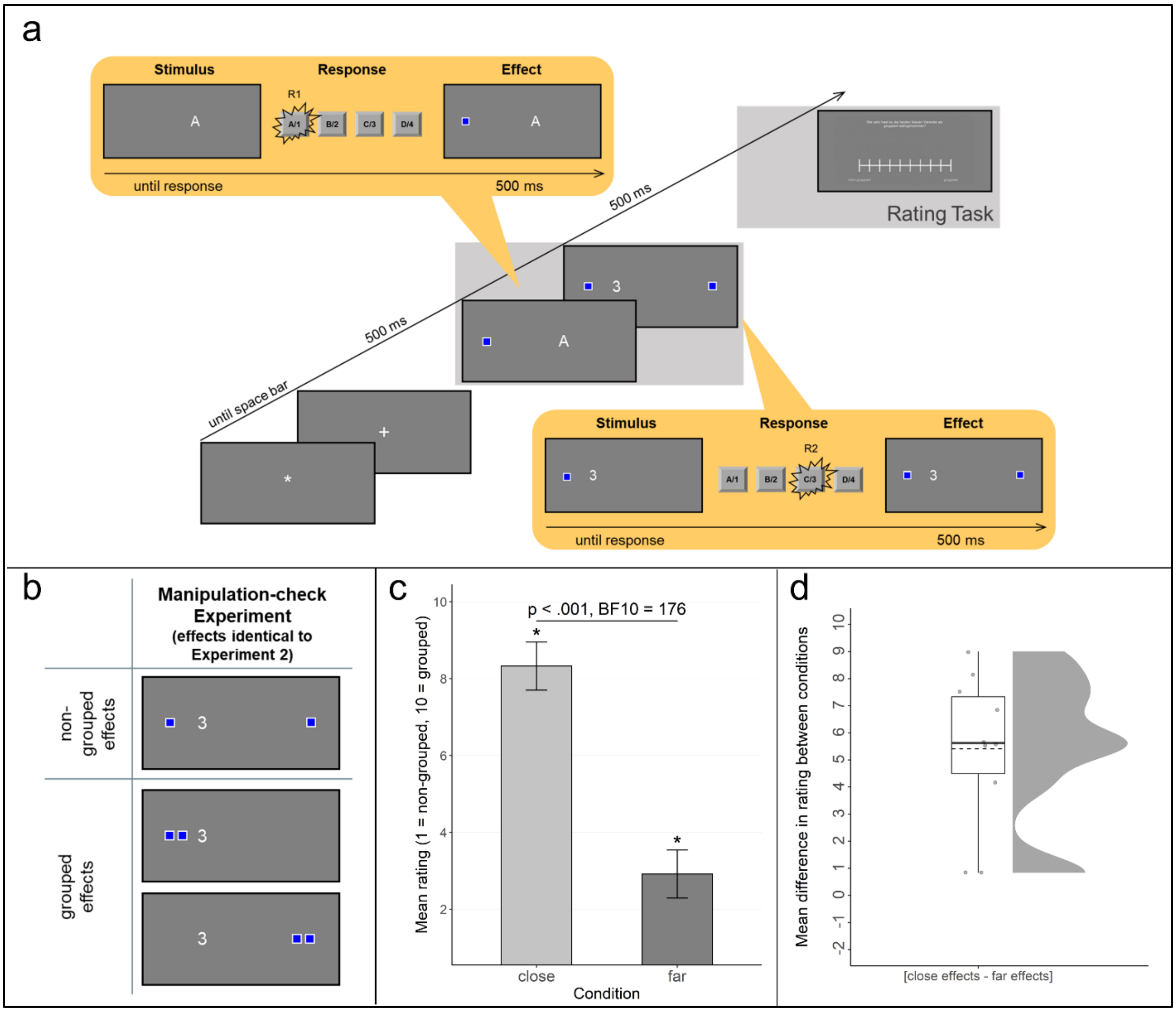

Figure A1. (a) Sequence of events in the Manipulation check experiment in one example trial. Participants gave two successive responses, R1 and R2, followed by a rating task. The stimuli and effects are not drawn to scale. (b) Effect positions depending on effect grouping condition. (c) Mean rating of perceived grouping of response effects on a 10-point rating scale (with 1 = "non-grouped" and 10 = "grouped") for spatially close vs. far effects. (d) Difference in mean rating between effect grouping conditions (calculated as [close effects – far effects] for each participant). Solid lines indicate the medians, dashed lines the means.

In sum, results from the rating task indicate that the majority of participants described the effects as being more strongly grouped in the spatially close condition than in the far condition.

APPENDIX B: Perceived response-effect relation

Table B1. Manipulation check questionnaire used in Experiment 2. Items 1-4 were of forced choice format, answers to items 5-6 were given on a 7-point rating scale (1: strongly disagree, 7: strongly agree). Results given as frequency or mean(SD) respectively. Items were translated to English (original: German).

Items	*Frequency*		*Mean(SD)*
	Yes	*No*	
1. Keypress answers and blue squares are related.	22	4	
2. Blue squares only lit up after a keypress.	19	7	
3. Blue squares always lit up, when I answered correctly.	23	3	
4. I used the blue squares as feedback for whether I answered correctly or not.	19	7	
5. Correct answers triggered blue squares to light up.			5.88(0.95)
6. Lighting up of blue squares was a consequence of correct answers			5.62(1.55)
